# Anti-atherosclerotic effects of naringenin and quercetin from Folium Artemisiae argyi by attenuating Interleukin-1 beta (IL-1β)/ matrix metalloproteinase 9 (MMP9): network pharmacology-based analysis and validation

**DOI:** 10.1186/s12906-023-04223-1

**Published:** 2023-10-25

**Authors:** Lei Zhang, Zhihui Yang, Xinyi Li, Yunqing Hua, Guanwei Fan, Feng He

**Affiliations:** 1 Hubei Key Laboratory of Economic Forest Germplasm Improvement and Resources Comprehensive Utilization, Huanggang Normal University, Huanggang, 438000 China; 2 Hubei Collaborative Innovation Center for the Characteristic Resources Exploitation of Dabie Mountains, Huanggang Normal University, Huanggang, 438000 China; 3 First Teaching Hospital of Tianjin University of Traditional Chinese Medicine, Tianjin, China; 4 Tianjin Key Laboratory of Translational Research of TCM Prescription and Syndrome, Tianjin, China

**Keywords:** Folium Artemisiae argyi, Atherosclerotic cardiovascular disease, Network pharmacology, Anti-inflammation, Quercetin, Naringenin

## Abstract

Effective components and related target genes of Folium Artemisiae argyi were screened from Traditional Chinese Medicines for Systems Pharmacology Database and Analysis Platform. The therapeutic targets of atherosclerosis were searched in the MalaCards and OMIM databases. Kyoto Encyclopedia of Genes and Genomes (KEGG) pathway analysis were performed in WebGestalt online and verified according to ClueGo and Pedia apps in Cytoscape. Then, the protein-protein interaction network was analyzed using the STRING database and constructed using Cytoscape. Differential expression of target genes was identified in GSE9128 and GSE71226 by GEO2R. And then, molecular docking was performed using the Molecular Operating Environment. Finally, we validated the protein expression of Interleukin-6 (IL-6)/IL-1β /MMP9 by qRT-PCR and Western blot in Raw264.7 which was induced by LPS. A total of 232 potential target genes and 8 ingredients of Folium Artemisiae argyi were identified. Quercetin and naringenin are potential candidate bioactive agents in treating atherosclerosis. Vascular endothelial growth factor (VEGFA), MMP9 and IL-1β could be potential target genes. KEGG analysis demonstrated that the fluid shear stress and atherosclerosis pathway play a crucial role in the anti-atherosclerosis effect of Folium Artemisiae argyi. Gene Expression Omnibus (GEO) validation demonstrated that VEGFA was downregulated, while MMP9 and IL-1β were upregulated in patients with atherosclerosis. Molecular docking suggested that only MMP9 had a good combination with quercetin. The cell experiment results suggested that naringenin and quercetin have strong anti-inflammation effects, and significantly inhibit the expression of MMP9.

**Practical Applications**

Artemisiae argyi is a traditional Chinese herbal medicine that has been widely used for its antibacterial and anti-inflammatory effects. This research demonstrated the bioactive ingredients, potential targets, and molecular mechanism of Folium Artemisiae argyi in treating atherosclerosis. It also suggests a reliable approach in investigating the therapeutic effect of traditional Chinese herbal medicine in treating Atherosclerotic cardiovascular disease (ASCVD).

**Supplementary Information:**

The online version contains supplementary material available at 10.1186/s12906-023-04223-1.

## Background

Atherosclerotic cardiovascular disease is a common pathology present in many cardiovascular diseases, arising from the obstruction of coronary vessels due to atherosclerosis or thrombosis. Cardiovascular diseases (CVDs) were the leading cause of death in non-communicable diseases according to the World Health Organization over the past decades [1]. CVD and stroke have become the two greatest causes of burden of disease in high-income countries. The risk factors of CVD vary and include sex, smoking, alcohol intake, and deficiencies in social relationships [2]. The causes of utmost concern in the general population are dyslipidemia and inflammation. Thus, statins and other drugs are widely used to reduce lipid levels and inflammation in hypercholesterolemia and other CVDs [3]. Although the effect is obvious, the side effects in some patients are also unavoidable [4]. The use of Traditional Chinese Medicine combination might be a potential supplementary treatment.

Artemisiae argyi is a Chinese herbal medicine containing many bioactive compounds, such as flavonoids, glycosides, sterols, and essential oils [5]. It has been widely used for the treatment of infections, cancers, and other inflammatory diseases [6]. Inflammation is also a major cause of CVDs [7]. Therefore, we explored the possible treatment of ASCVD using Artemisia argyi according to a pharmacology-based network analysis method [8].

Network pharmacology [9] is a novel paradigm that integrates the concepts of network science and pharmacology, provides numerous valuable advantages in the research of drug discovery and development. The traditional medicine pharmacology network prediction analysis is a method involving the pharmacogenomics and therapeutic mechanism of traditional Chinese medicinal herbs and/or formulae and the potential target genes and/or drugs [10]. The comprehensive investigation of the relationships among drugs, target genes, and diseases are possible because of the rapid development of bioinformatics and pharmacology [11].

## Methods

### Compounding ingredients of Folium Artemisiae argyi

Compounds from Folium Artemisiae argyi were determined using the public databases Traditional Chinese Medicines for Systems Pharmacology Database and Analysis Platform (TCMSP, https://tcmspw.com/tcmsp.php) [12] and Integrative Pharmacology-based Research Platform of Traditional Chinese Medicine (TCMIP, http://www.tcmip.cn/TCMIP) [13].

### Pharmacokinetic absorption, distribution, metabolism, and excretion (ADME) screen

The ADME criteria of Folium Artemisiae argyi were extracted from the TCMSP database. Drug-likeness (DL) and oral bioavailability (OB) were selected to identify the bioactive ingredients of Folium Artemisiae argyi. OB is the percentage of an oral dose capable of producing pharmacological activity [14]. DL is an indicator for determining the similarity or likeness of a compound that can help in determining whether a compound has a therapeutic effect or not [15].

### Targets of compounds searching

Information on the compounded ingredient target genes was obtained from the TCMSP database, and the Drug Bank [16] (https://go.drugbank.com/) database was also used for determining the comprehensive drug targets of all ingredients. The related target genes of atherosclerosis were searched from the Mala Cards (https://www.malacards.org/) and OMIM (https://omim.org/) databases. The target genes of compounds were collected according to the Similarity ensemble approach (SEA) [17] online database (http://sea.bkslab.org/).

### Protein-protein interaction (PPI) network

The overlapping genes of AS and the compounds were selected as the hub genes and analyzed using the database STRING [18] (https://string-db.org), which could provide the PPI network results. The Cytoscape [19] (https://cytoscape.org/) software is widely applied to pharmacology studies in network construct and visualization.

### KEGG analysis and enrichment

KEGG database was established by the Kanehisa Laboratory in 1995 and is typically used in pathway analysis and annotation in network pharmacology. We used WebGestalt [20] (WEB-based Gene Set Analysis Toolkit, http://www.webgestalt.org/) for KEGG pathway analysis, which is a functional enrichment analysis web tool. Then, the interactions between genes and pathways were validated by ClueGo and Pedia apps in Cytoscape.

### GEO validation

Candidate target genes were identified in the GEO database (GSE9128, GSE71226), including total RNA expression data from coronary heart disease in human. GEO2R was used to identify the differentially expressed genes (DEGs), p ≤ 0.05, and |log FC| > 1 were the screening limitations [21].

### Molecular docking

Molecular docking is a crucial technology in proteins and small compounds. It is performed using the Molecular Operating Environment (MOE, v2019.0102) software to validate interactions between compounds and target proteins. The 3D structure of targets was obtained from the Protein Data Bank (PDB, http://www.rcsb.org) and imported into MOE to perform molecular docking after protein structure preparation. The structure of participant compounds was obtained from PubChem (https://pubchem.ncbi.nlm.nih.gov).

### Cell culture and treatment

Raw264.7 was provided by Tianjin University of Traditional Chinese Medicine, and cultured by Dulbecco’s modified Eagle medium (DMEM) containing 10% fetal bovine serum and 1% penicillin/streptomycin in an incubator (5% CO2, 37^o^C). The cells were stimulated with or without lipopolysaccharide (LPS) (10 µg/ml) in the presence or absence of quercetin (10, 20, 50 µM), naringenin (10, 20, 50 µM).

### Real-time quantitative reverse transcription PCR and western blot analysis

The total RNA of Raw264.7 was isolated using an RNA extraction kit (Vazyme Biotech Co., Ltd), according to the manufacturer’s instructions. The concentration of extracted RNA was detected using NanoDrop (Thermo), and complementary DNA (cDNA) was synthesized according to the manufacturer’s instructions of RNA reverse transcription kit (Thermo). The messenger RNA (mRNA) expression levels of Interleukin-6 (IL-6), Interleukin-1 beta (IL-1β), matrix metallopeptidase 9 (MMP9) were analyzed using quantitative real-time polymerase chain reaction (qRT-PCR) on the LightCycler 96 (Roche) with SYBR Green (Thermo). Relative expression was calculated as 2^−ΔΔCt^ using glyceraldehyde 3-phosphate dehydrogenase (GAPDH) as a reference gene. Primers were purchased from Sangon Biotech (Shanghai, China), sequences were listed in Table 1. Protein expression of IL-6/ IL-1β/ MMP9 were determined by Western blot. Rabbit anti-IL-6 (21865-1-AP) Polyclonal antibody was purchased from Proteintech; Mouse anti- IL-1β (SC-52,012), MMP-9 (SC-393,859) monoclonal antibody were purchased from Santa Cruz Biotechnology, Inc. (Santa Cruz, CA); Mouse anti-β-actin monoclonal antibody were purchased from Cell Signaling Technology, Inc. (Danvers, MA, USA). Quercetin and naringenin were purchased from Yuanye (Shanghai, China).

**Table 1:** Primers of RT-PCR

Gene name	Sequence
MMP9-F	CTGGACAGCCAGACACTAAAG
MMP9-R	CTCGCGGCAAGTCTTCAGAG
IL-1β-F	GAAATGCCACCTTTTGACAGTG
IL-1β-R	TGGATGCTCTCATCAGGACAG
IL-6 -F	CTGCAAGAGACTTCCATCCAG
IL-6 -R	AGTGGTATAGACAGGTCTGTTGG
GAPDH- F	TGACCTCAACTACATGGTCTACA
GAPDH-R	CTTCCCATTCTCGGCCTTG

### Data analysis

All data analysis were proceeding according online database (https://tcmspw.com/tcmsp.php, https://go.drugbank.com/, https://www.malacards.org/, https://omim.org/, http://sea.bkslab.org/, https://string-db.org, http://www.webgestalt.org/, https://www.ncbi.nlm.nih.gov/geo/geo2r/) and MOE software (v2019.0102). Statistical analysis was performed by GraphPad (PRISM 7.0.a), statistical significance was considered as p < 0.05, the differences among groups were analyzed with one-way ANVOA .

## Results

### Compounding ingredients of Folium Artemisiae argyi

**Fig. 1:**
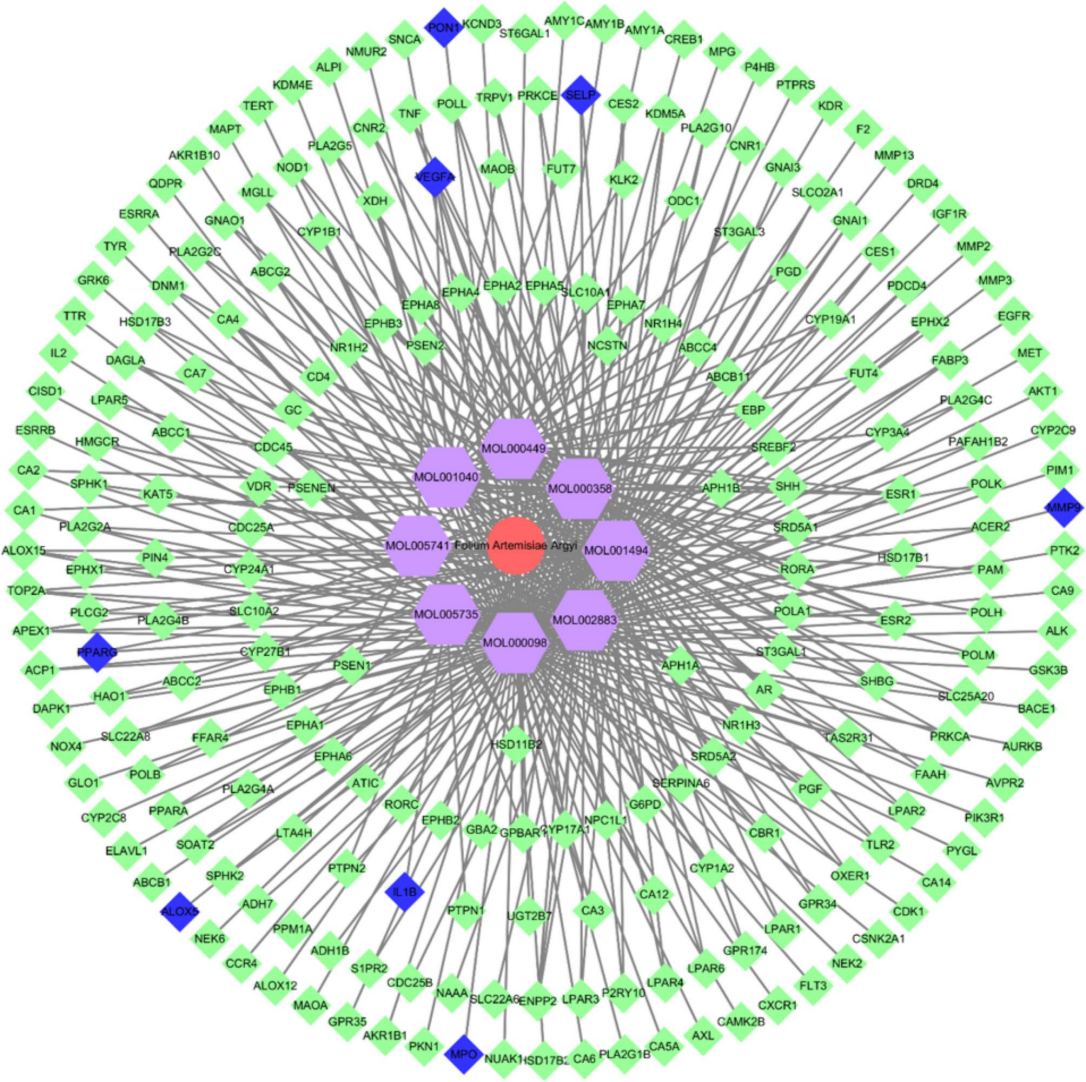
Protein-protein interaction network of common targets of Folium Artemisiae argyi and atherosclerosis. Red circle: Folium Artemisiae argyi; Purple hexagon: ingredients of Folium Artemisiae argyi after screening by OB ≥ 30% and DL ≥ 0.18; green diamonds: target genes (human) of the ingredients; blue diamonds: eight hub genes overlapped among “atherosclerosis-related genes” from the Malagenes and OMIM databases and “compounding ingredients target genes.”

We input “Folium Artemisiae Argyi” as an “herb name” to search the ingredients of the compound. A total of 135 items were obtained, and only 9 ingredients were included after screening by OB ≥ 30% and DL ≥ 0.18 in this study (Table 2). The target genes of the nine ingredients were collected from the SEA (Similarity ensemble approach, http://sea.bkslab.org/) database, which is a database that can be searched for chemical formulas according to their ingredients. After selecting genes from humans and eliminating the duplicate genes, 8 ingredients and 232 genes were included. The network of compounds to target genes was constructed using Cytoscape (Fig. 1).

**Table 2:** Compounding ingredients of Folium Artemisiae argyi (TCMSP)

Mol ID	Molecule Name	OB (%)	DL	Compound CID (PubChem)
MOL002883	ethyl oleate (NF)	32.4	0.19	5363269
MOL000358	beta-sitosterol	36.91	0.75	222284
MOL005741	cycloartenol acetate	41.11	0.8	13023741
MOL005720	24-methylenecyloartanone	41.11	0.79	none
MOL001494	mandenol	42	0.19	5282184
MOL001040	(2R)-5,7-dihydroxy-2-(4-hydroxyphenyl)chroman-4-one	42.36	0.21	667495
MOL000449	stigmasterol	43.83	0.76	5280794
MOL005735	dammaradienyl acetate	44.83	0.83	14137679
MOL000098	quercetin	46.43	0.28	5280343

### Atherosclerosis-related target genes

**Fig. 2:**
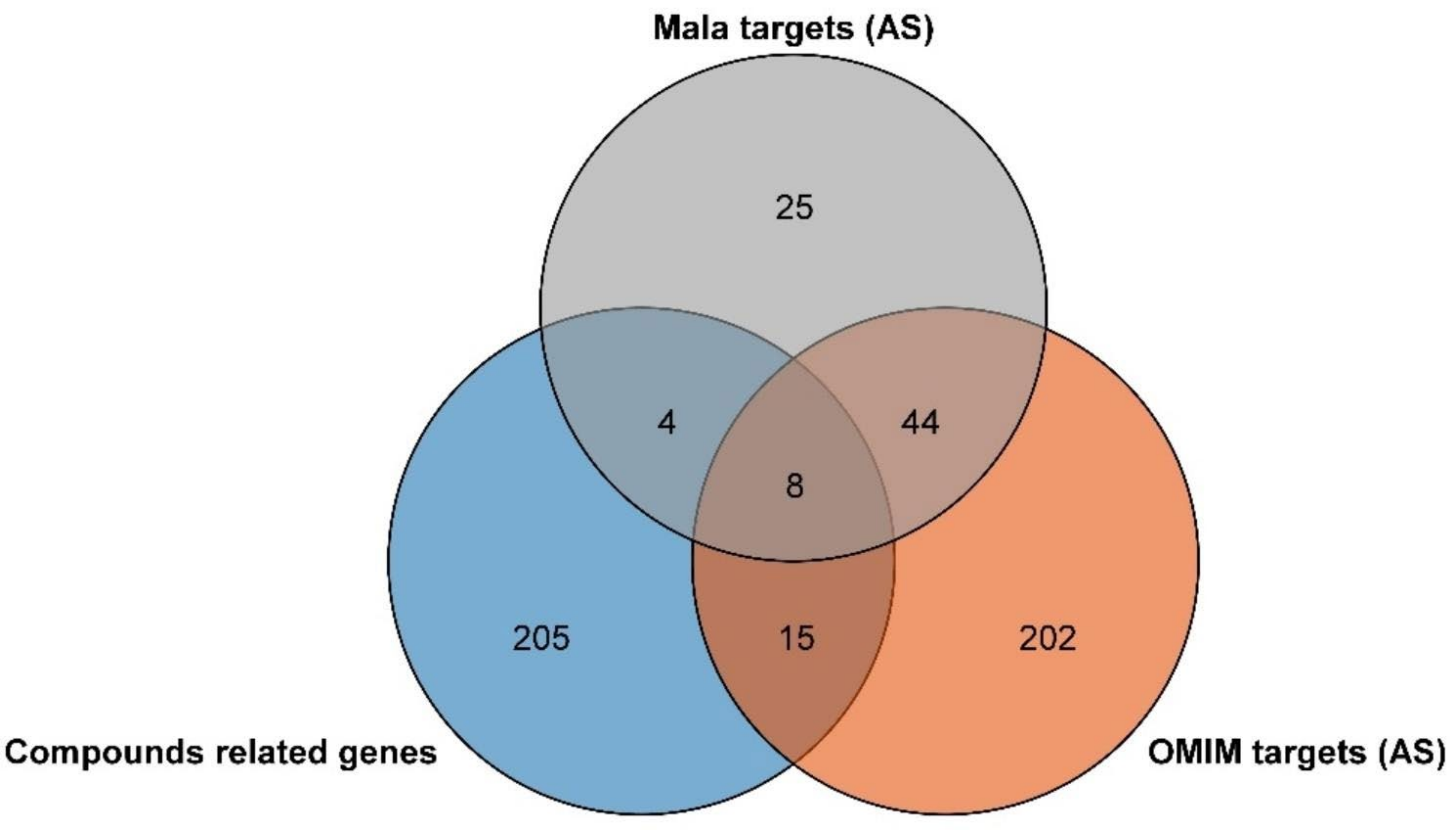
The Venn diagram revealed the overlapping genes among “atherosclerosis-related genes” from Mala genes and OMIM database and the “compound ingredients target genes”. Gray circle: “atherosclerosis-related genes” from the Mala genes database; orange circle: “atherosclerosis-related genes” from the OMIM database; and blue circle: “compounding ingredients target genes” from the SEA database

We searched the Mala Cards database online using the keyword “atherosclerosis” and 81 target genes were selected. We searched the Online Mendelian Inheritance in Man (OMIM) database with the same keywords, and 269 genes were collected. The overlapping genes among “atherosclerosis-related genes” from Mala genes and OMIM database and the “compound ingredients target genes”; finally, eight hub genes of the nodes with the highest connections that appear in the network were identified (Fig. 2).

### Network construction of protein-protein interaction (PPI)

**Fig. 3:**
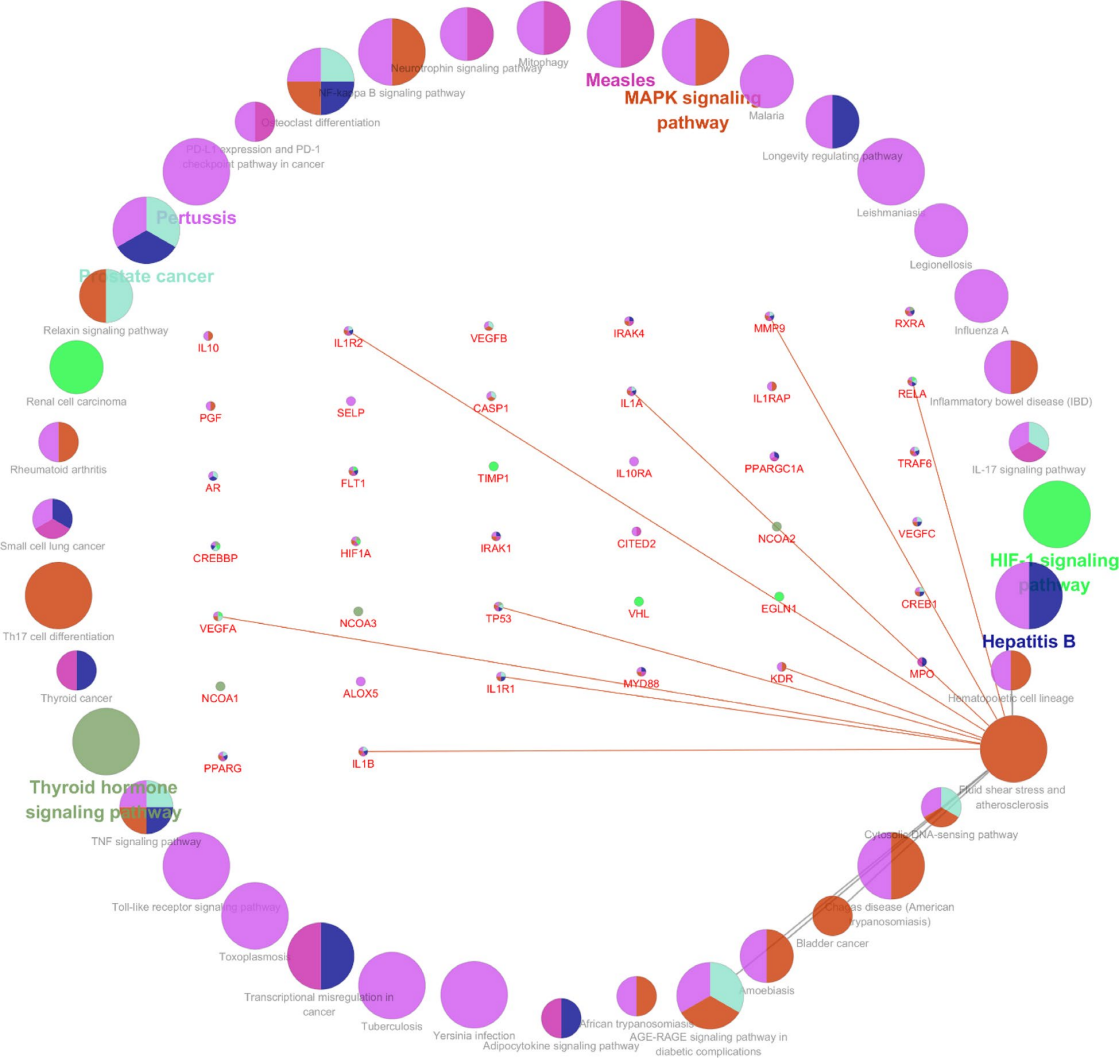
Network of the main pathways and targets of Folium Artemisiae argyi treatment of atherosclerosis. “Fluid shear stress and atherosclerosis” pathway [39] and related genes. in the center of the circle were the genes related “Fluid shear stress and atherosclerosis” (linked with red lines)

**Fig. 4:**
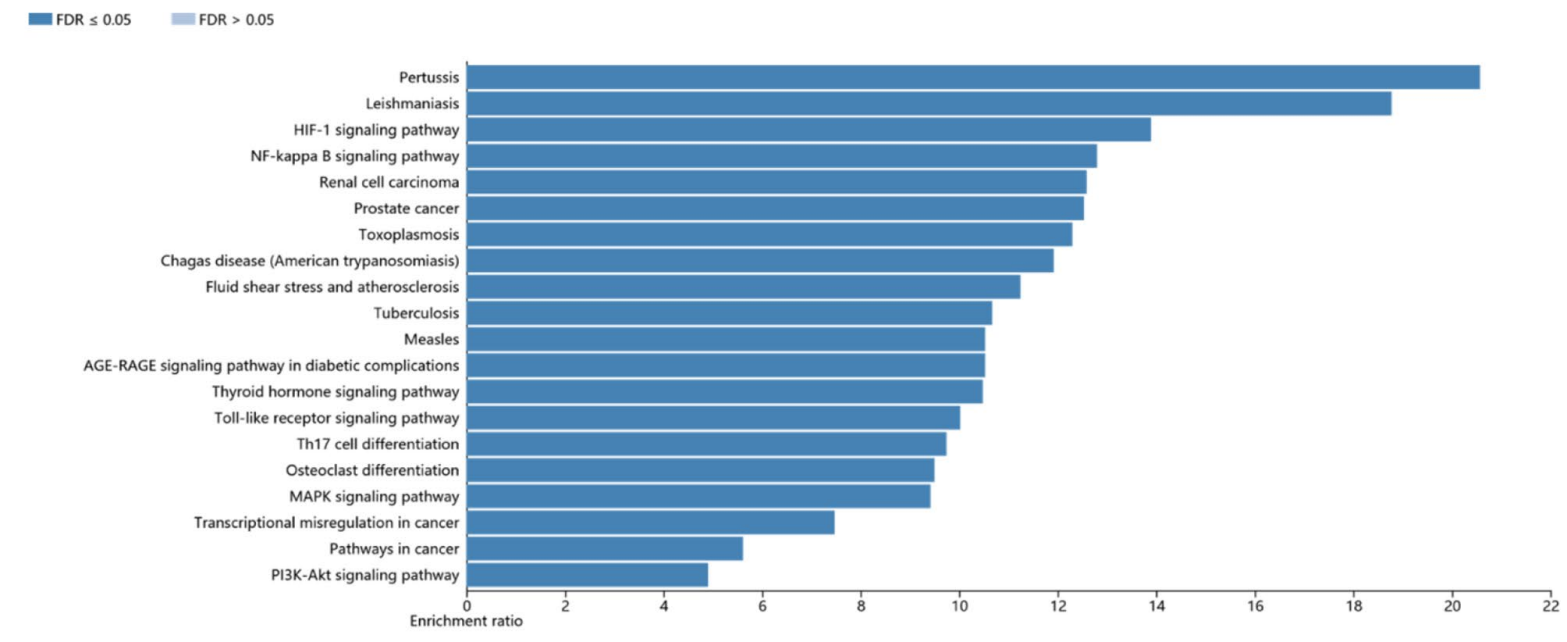
The KEGG pathway enrichment analysis results. Top 20 pathways analyzed in WebGestalt, arranged by the enrichment ratio from high to low

The eight hub genes were input into the online tool “STRING”, and the PPI network was constructed by the limitation: “minimum required interaction score, (confidence = 0.500); max number of interactors, (1st shell ≤ 20 interactors, 2nd shell ≤ 20 interactors)”; in total, 48 interactors were collected. Visualization of common targets and related signaling pathways was created, larger node in the graph means higher degree value and greater likelihood to the targets (Fig. 3). KEGG pathway analysis of the 48 genes was performed using the online web tools WebGestalt. The top 20 pathways are listed in Table 3; Fig. 4. The results were validated using ClueGo + Pedia apps, and the “Fluid shear stress and atherosclerosis” pathway (Fig. 3), including the three genes (*IL-1β, MMP9, VEGFA*), were the target genes in the eight hub genes.

**Table 3:** KEGG pathway analysis in WebGestalt (Top 20)

GeneSet	Description	P Value	FDR
hsa04010	MAPK signaling pathway	2.37E-12	7.73E-10
hsa05133	Pertussis	3.07E-10	5.01E-08
hsa05200	Pathways in cancer	1.51E-09	1.64E-07
hsa05152	Tuberculosis	3.25E-09	2.65E-07
hsa05140	Leishmaniasis	6.93E-09	4.52E-07
hsa05418	Fluid shear stress and atherosclerosis	6.81E-08	3.57E-06
hsa04066	HIF-1 signaling pathway	7.67E-08	3.57E-06
hsa05145	Toxoplasmosis	2.00E-07	8.14E-06
hsa05162	Measles	6.63E-07	2.40E-05
hsa04064	NF-kappa B signaling pathway	9.56E-07	3.12E-05
hsa05215	Prostate cancer	1.10E-06	3.26E-05
hsa05142	Chagas disease (American trypanosomiasis)	1.55E-06	4.21E-05
hsa04919	Thyroid hormone signaling pathway	3.69E-06	9.25E-05
hsa04380	Osteoclast differentiation	7.11E-06	1.65E-04
hsa05202	Transcriptional misregulation in cancer	8.75E-06	1.90E-04
hsa04933	AGE-RAGE signaling pathway in diabetic complications	1.91E-05	3.89E-04
hsa04151	PI3K-Akt signaling pathway	2.38E-05	4.56E-04
hsa04620	Toll-like receptor signaling pathway	2.53E-05	4.58E-04
hsa04659	Th17 cell differentiation	2.98E-05	5.11E-04
hsa05211	Renal cell carcinoma	4.26E-05	6.94E-04

### GEO validation

Analysis of GSE71226 and GSE9128 expression data of atherosclerosis group revealed that *VEGFA* was downregulated, while *MMP9* and *IL-1β* were upregulated (Table 4, Supplementary Tables 1 and 2). Thus, the three genes might be the candidate therapeutic targets of Folium Artemisiae argyi in the clinical treatment of atherosclerosis.

**Table 4:** GEO validation using GEO2R

Expression	Gene symbol	Gene title	P Value	log FC
upregulated genes	IL-1β	interleukin 1 beta	0.00933762	1.20182753
	MMP9	matrix metallopeptidase 9	0.0187166	2.267172
downregulated gene	VEGFA	vascular endothelial growth factor A	0.0011307	-1.6941442

### MOE docking

**Figs. 5:**
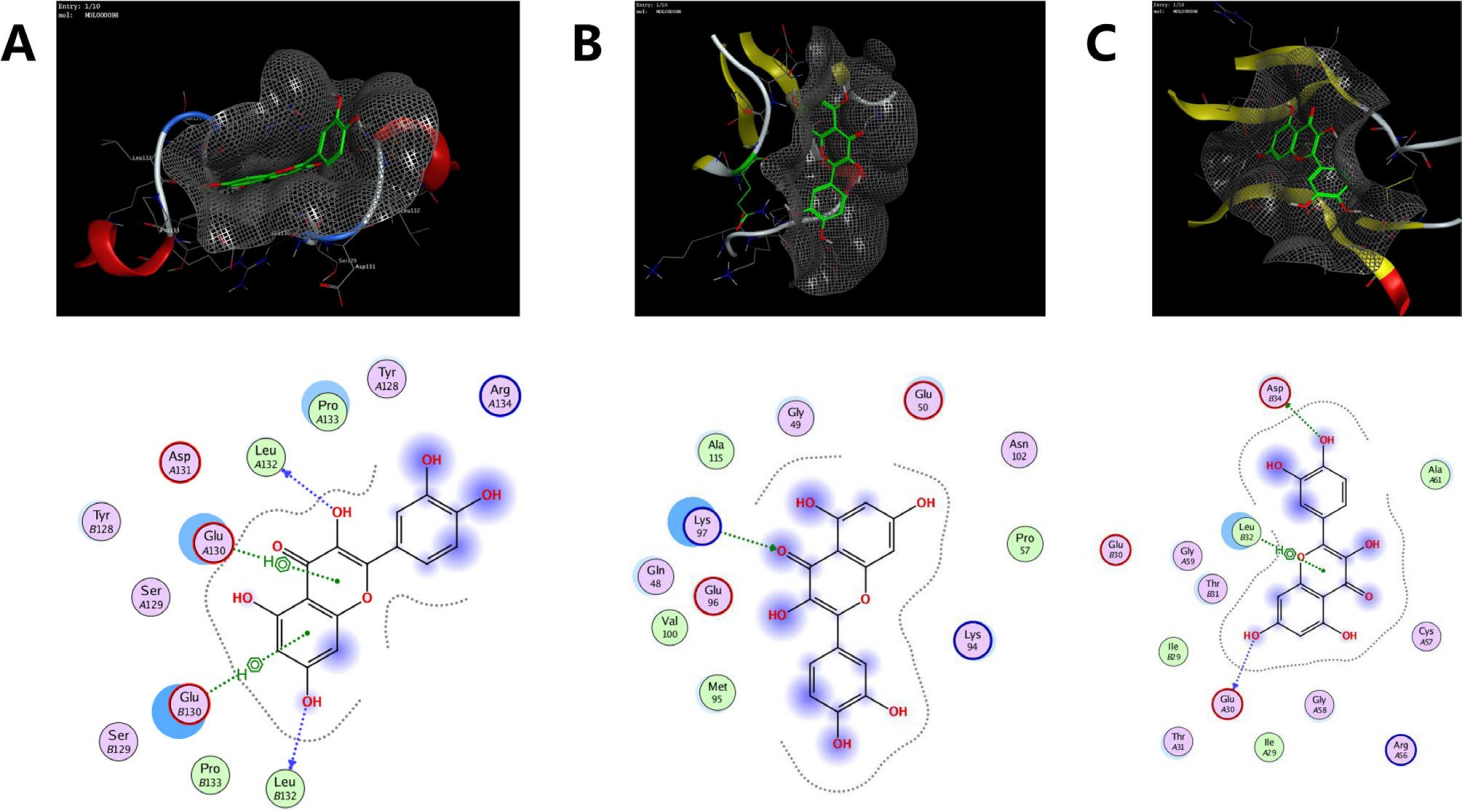
Molecular docking results of the main compounds and protein targets. (**A-C**) represent quercetin interacted action mode with MMP9 (**A**), IL-1β (**B**), VEGFA (**C**)

**Figs. 6:**
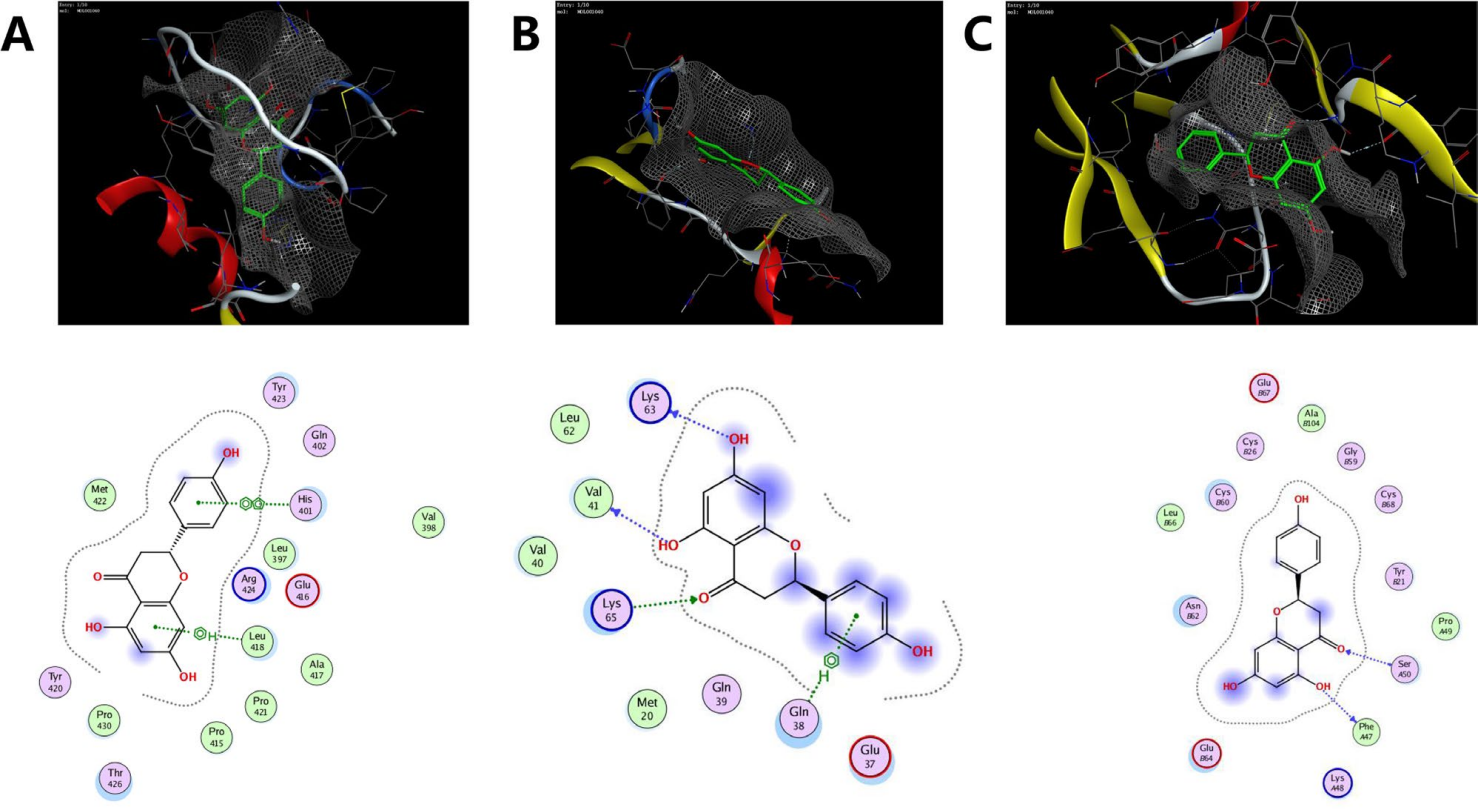
Molecular interactions between the main compounds and protein targets. (**A-C**) represent naringenin interacted action mode with MMP9 (**A**), IL-1β (**B**), VEGFA (**C**)

Molecular docking was performed to validate the interaction of the target protein IL-1β (PDB code: 2nvh), MMP9 (PDB code: 2ow1), and VEGFA (PDB code: 1mkk) and the related participant compounds (MOL000098, and MOL001040). Visualization of docking results of receptors and ligands were presented in Figs. 5 and 6. The results of Fig. 5A-C showed that quercetin could interact with MMP9, IL-1β, VEGFA. Figure 5A shows that quercetin can interact with Leu132 and Glu130 in MMP9, Fig. 5B shows quercetin interact with Lys97 in IL-1β, and Fig. 5C shows quercetin interact with Asp34, Leu32 and Glu30 in VEGFA. Figure 6 A represent naringenin interact with His401 and Leu418 in MMP9, Fig. 6B shows naringenin interact with Lys63, Lys65 and Gln38 in IL-1β, Fig. 6C shows naringenin interact with Ser50 and Phe47 in VEGFA. The binding energy score is shown in Table 5. Lower binding energy means higher affinity between the receptor and the ligand, and the conformation is more stable. Generally, binding energy which is less than − 5 kcal/mol indicates a good binding activity [22]. The molecular docking results predicted that quercetin and naringenin from Folium Artemisiae argyi could be effective in atherosclerosis therapy by targeting MMP9.

**Table 5:** The binding energy score of ingredients with MMP9, IL-1β and VEGFA s (Kcal/Mol).

Receptors	Binding energy score	
	quercetin	naringenin
MMP9	-6.0273	-6.6075
IL-1β	-5.1048	-5.3381
VEGFA	-5.4751	-5.5095

### Quercetin and naringenin suppressed LPS-induced pro-inflammatory cytokines

**Fig. 7:**
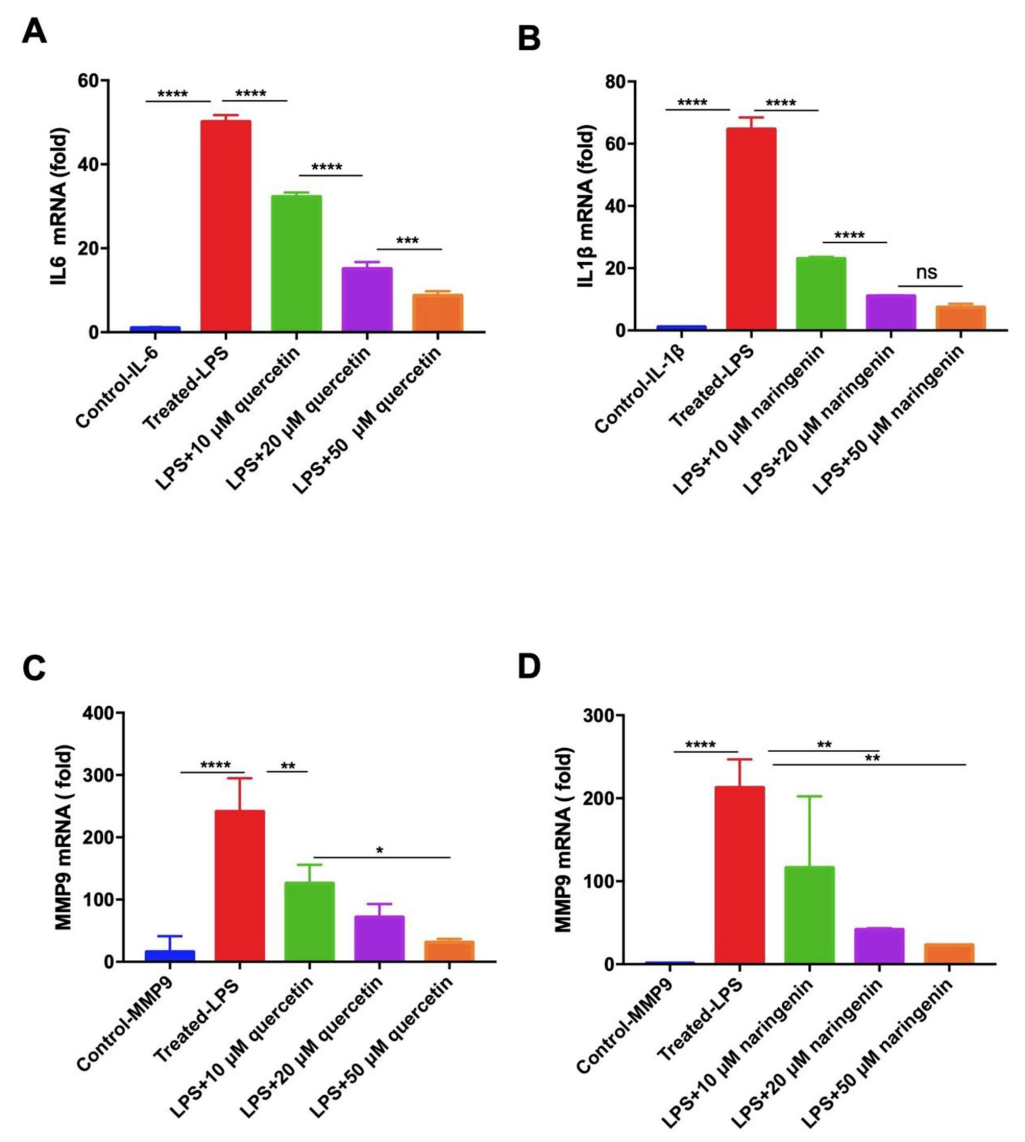
The qRT-PCR results, mRNA expression of IL6, IL-1β and MMP9 in macrophage (RAW264.7 stimulated with LPS) treated with quercetin and naringenin. The anti-inflammation effect of quercetin and naringenin indicated MMP9 might be the potential target. LPS: lipopolysaccharide. * P < 0.05,** P < 0.01,*** P < 0.001, **** P < 0.0001. (**A**) Control-IL-6 vs. Treated-LPS P < 0.0001,Treated-LPS vs. LPS+10 μM quercetin P < 0.0001, LPS+10 μM quercetin vs. LPS+20 μM quercetin P < 0.0001, LPS+20 μM quercetin vs. LPS+50 μM quercetin P = 0.0006 (**B**) Control-IL-1β vs. Treated-LPS P < 0.0001, Treated-LPS vs. LPS+10 μM naringenin P < 0.0001, LPS+10 μM naringenin vs. LPS+20 μM naringenin P < 0.0001, LPS+20 μM naringenin vs. LPS+50 μM naringenin P = 0.1643 (**C**) Control-MMP9 vs. Treated-LPS P < 0.0001, Treated-LPS vs. LPS+10 μM quercetin P = 0.0076, LPS+10 μM quercetin vs. LPS+50 μM quercetin P = 0.0266 (**D**) Control-MMP9 vs. Treated-LPS P = 0.0007, Treated-LPS vs. LPS+20 μM naringenin P = 0.0035, Treated-LPS vs. LPS+50 μM naringenin P = 0.0017

**Fig. 8:**
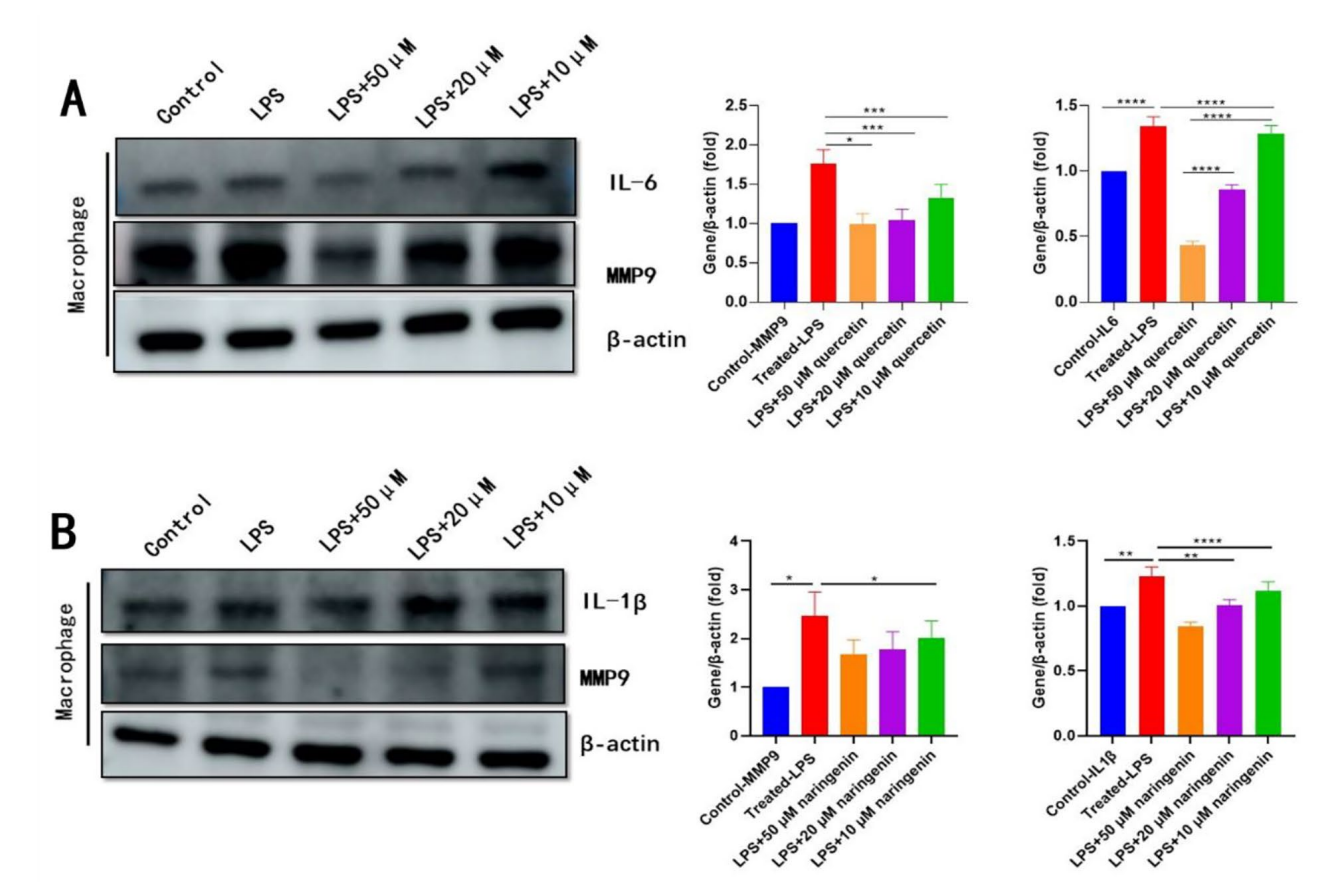
Western blotting results, protein expression of IL6, IL-1β and MMP9 in macrophage (RAW264.7 stimulated with LPS) in each group treated with quercetin (**A**) and naringenin (**B**). * P < 0.05,** P < 0.01,*** P < 0.001, **** P < 0.0001. (Original blot were included in the “Supplementary material file”)

To investigate the effects of quercetin and naringenin on anti-inflammation, Raw264.7 were stimulated with LPS in the presence or absence of quercetin (10, 20, 50 µM) and naringenin (10, 20, 50 µM) for 24 h. As shown in Figs. 7 and 8, the mRNA and protein expression of IL-6 and IL-1β were significantly increased with the LPS treatment (P < 0.0001), which were inhibited by quercetin (10, 20, 50 µM) and naringenin (10, 20, 50 µM). These results provided evidence that quercetin and naringenin have a strong inhibitory effect on pro-inflammatory cytokines.

### MMP9 might be a therapeutic target of Folium Artemisiae argyi in ASCVD

The mRNA and protein expression level of MMP9 had a significant increase after treatment with LPS in the Raw264.7, quercetin and naringenin could significantly decrease the mRNA and protein expression level of MMP9 (Fig. 7 C, D and Fig. 8), suggesting that lowering the expression of MMP9 might be the therapeutic effect of quercetin and naringenin in the treatment of ASCVD.

## Discussion

Multiple factors are associated with cardiovascular diseases [23]; a high low-density lipoprotein cholesterol (LDL-C) concentration of plasma and inflammation are the major factors that cause atherosclerosis. Lowing cholesterol uptake and increasing cholesterol efflux could attenuating the inflammation of atherosclerosis plaque and even promote plaque regression [24]. Although statin treatment in lowering LDL-C has achieved a relatively optimistic result, its benefits are limited by adverse effects to the liver and others, and further effective drugs for treating ASCVD should be sought. Inflammation plays an important role in theprocess of atherosclerosis, the Canakinumab Anti-inflammatory Thrombosis Outcomes Study (CANTOS) showed that targeting inflammation in atherosclerosis in clinic was effective [25].

In the present work, we constructed a network of bioactive compounds and the molecular targets of Folium Artemisiae argyi that overlapped the target genes between atherosclerosis and related ingredients of Folium Artemisiae argyi. Finally, eight hub genes were identified, and *IL-1β, VEGFA*, and *MMP9* genes in the fluid shear stress and atherosclerosis pathway are the most likely target genes in treating atherosclerosis. The results of the GEO database (GSE71226, GSE9128) validation revealed that IL-1β and MMP9 expression was upregulated, and VEGFA was downregulated significantly compared with controls. The expression values of VEGFA from GSE9128 in the control group were higher compared to the Ischemic cardiomyopathy (ICM) group, coincident with early research that increased the expression of VEGFA might be a potential therapeutic method for ICM [26].

MMP9 involved in the matrix-metalloproteinases family has been implicated in regulating matrikines. Given their ability to alter cellular migration and mitogenesis, matrikines have been implicated in inflammation, wound repair, and atherosclerosis [27]. MMP9 plays a role in inflammation and is upregulated in a lipopolysaccharide (LPS) model of corneal inflammation [28]. And the molecular docking results also suggested that MMP9 has better interactivity with quercetin, the experiment results in Raw264.7 were also providing evidence that quercetin and naringenin could decrease the expression of MMP9 and suppressed the expression of pro-inflammation cytokines IL-6 and IL-1Β.

In our study, the LPS induced inflammation in Raw264.7 also elevated the mRNA and protein expression level of MMP9, as while treated with quercetin (10, 20, 50 µM) and naringenin (10, 20, 50 µM) could significantly decrease its expression (Fig. 7 C, D and Fig. 8). And these results suggested quercetin might have the effect of steady atherosclerotic plaque stability by inhibiting MMP9 expression.

IL-1β is a member of the IL-1 family cytokines; it is an immunomodulatory signaling molecule and thus acts as a central mediator [29]. The Canakinumab Anti-Inflammatory Thrombosis Outcome Study trial also provided proof for the inflammation hypothesis of atherosclerosis [30], and IL-1β inhibition highlighted the potential of anti-inflammatory therapies to improve the clinical outcomes of CVDs. The results of our research also presented the suppression of quercetin and naringenin to pro-inflammation cytokines in IL-1β and IL6 (Figs. 7A and B and 8).

The MMP9 and IL-1β-related major ingredients of Folium Artemisiae argyi were quercetin and naringenin. Quercetin, one of the ingredients of Folium Artemisiae argyi, has shown a wide range of biological actions in anti-inflammatory and antiviral activities in vitro and in some animal models [31]. The ability of inflammation to promote atherosclerosis has been elucidated in molecular and cellular pathways by numerous experimental works [32]. Quercetin is a kind of flavonoid, and a prominent dietary antioxidant present in fruits, vegetables, and herbal medicines, it plays a role in attenuating atherosclerosis by alleviating inflammation and improving nitric oxide (NO) bioavailability [33].

Naringenin is also one of the natural flavanones in Folium Artemisiae argyi, and animal models have demonstrated its therapeutic potentials in treating inflammation-related diseases, such as atherosclerosis [34]. Naringenin suppresses inflammatory cytokine production during transcription and post-transcription; it not only inhibits cytokine mRNA expression but also promotes lysosome-dependent cytokine protein degradation [35]. Thus, the anti-atherosclerotic activity of naringenin is due to its high anti-inflammatory effects [36].

Inflammation is an important driver of atherosclerosis, and cellular inflammatory changes actively contribute to atherosclerosis progression [37, 38]. The therapeutic effect of the inflammatory pathway targets helped improve the outcomes of patients with cardiovascular diseases. The anti-inflammatory effects of the ingredients from Folium Artemisiae argyi were obvious. Consequently, Folium Artemisiae argyi has potential beneficial effects in atherosclerosis therapy through its anti-inflammatory activities. However, our research has limitations in investigating the mechanism of Folium Artemisiae argyi used in treating atherosclerosis. And its application to clinical medicine in the future should be determined through extensive experiments in vivo and in vitro.

## Conclusions

In the present study, we performed network pharmacology-based prediction, molecular docking, and GEO database validation to verify the potential targets of Folium Artemisiae argyi through related bioactive ingredients in treating atherosclerosis. And the validation in the LPS-induced inflammation model of Raw264.7 also offered evidence that quercetin and naringenin have the anti-inflammation effect and suppressed the expression of MMP9. We demonstrated that the anti-inflammatory and keeping the atherosclerotic plaque stable ability of Folium Artemisiae argyi may be the main direction in atherosclerosis therapy in the future, which also provided a practicable application for the analysis of traditional Chinese medicine in the clinical treatment of diseases.

### Electronic supplementary material

Below is the link to the electronic supplementary material.

Supplementary Material 1

Supplementary Material 2

Supplementary Material 3

Supplementary Material 4

## Data Availability

The datasets used and/or analysed during the current study are available from the corresponding author on reasonable request. The data that support the findings of this study are openly available in [“figshare “] at 10.6084/m9.figshare.21916380.

## References

[1] Zhu K-F, Wang Y-M, Zhu J-Z (2016). National prevalence of coronary Heart Disease and its relationship with human development index: a systematic review. Eur J Prev Cardiol.

[2] Valtorta NK, Kanaan M, Gilbody S (2016). Loneliness and social isolation as risk factors for coronary Heart Disease and Stroke: systematic review and meta-analysis of longitudinal observational studies. Heart.

[3] Wang C-Y, Liu P-Y, Liao JK (2008). Pleiotropic effects of statin therapy: molecular mechanisms and clinical results. Trends Mol Med.

[4] Schmitz G, Langmann T (2006). Pharmacogenomics of cholesterol-lowering therapy. Vascul Pharmacol.

[5] Zhang LB, Lv JL, Chen HL (2013). Chemical constituents from Artemisia argyi and their chemotaxonomic significance. Biochem Syst Ecol.

[6] Adams M, Efferth T, Bauer R. Activity-guided isolation of Scopoletin and Isoscopoletin, the inhibitory active principles towards CCRF-CEM Leukaemia cells and Multi-drug Resistant CEM/ADR5000 cells, from Artemisia Argyi. Planta Med. 2006;72(9). 10.1055/s-2006-947165 16881019

[7] Hansson GK (2005). Inflammation, Atherosclerosis, and coronary artery Disease. N Engl J Med.

[8] Huang S, Zhang Z, Li W (2020). Network Pharmacology-Based Prediction and Verification of the active ingredients and potential targets of Zuojinwan for treating Colorectal Cancer. Drug Des Devel Ther.

[9] Hopkins AL (2008). Network pharmacology: the next paradigm in drug discovery. Nat Chem Biol.

[10] Zuo H, Zhang Q, Su S (2018). A network pharmacology-based approach to analyse potential targets of traditional herbal formulas: an example of Yu Ping Feng decoction. Sci Rep.

[11] Guo P, Cai C, Wu X (2019). An insight into the molecular mechanism of Berberine towards multiple Cancer types through systems Pharmacology. Front Pharmacol.

[12] Ru J, Li P, Wang J (2014). TCMSP: a database of systems pharmacology for drug discovery from herbal medicines. J Cheminform.

[13] Xu HY, Zhang YQ, Liu ZM (2019). ETCM: an encyclopaedia of traditional Chinese medicine. Nucleic Acids Res.

[14] Chen ML, Shah V, Patnaik R (2001). Bioavailability and bioequivalence: an FDA regulatory overview. Pharm Res.

[15] Kim SK, Lee S, Lee MK (2019). A systems pharmacology approach to investigate the mechanism of oryeong-san formula for the treatment of Hypertension. J Ethnopharmacol.

[16] Wishart DS, Feunang YD, Guo AC (2018). DrugBank 5.0: a major update to the DrugBank database for 2018. Nucleic Acids Res.

[17] Keiser MJ, Roth BL, Armbruster BN (2007). Relating protein pharmacology by ligand chemistry. Nat Biotechnol.

[18] Szklarczyk D, Kirsch R, Koutrouli M (2023). The STRING database in 2023: protein-protein association networks and functional enrichment analyses for any sequenced genome of interest. Nucleic Acids Res.

[19] Shannon P, Markiel A, Ozier O (2003). Cytoscape: a software environment for integrated models of biomolecular interaction networks. Genome Res.

[20] Wang J, Duncan D, Shi Z et al. WEB-based GEne SeT AnaLysis Toolkit (WebGestalt): update 2013. Nucleic Acids Res. 2013;41(Web Server issue):W77–83. 10.1093/nar/gkt439 PMC3692109 23703215

[21] Liang B, Xiang Y, Zhang X (2020). Systematic pharmacology and GEO database mining revealed the therapeutic mechanism of Xuefu Zhuyu Decoration for Atherosclerosis Cardiovascular Disease. Front Cardiovasc Med.

[22] Liu J, Liu J, Tong X (2021). Network Pharmacology Prediction and Molecular Docking-based strategy to Discover the potential pharmacological mechanism of Huai Hua San Against Ulcerative Colitis. Drug Des Devel Ther.

[23] Yusuf S, Hawken S, Ounpuu S (2004). Effect of potentially modifiable risk factors associated with Myocardial Infarction in 52 countries (the INTERHEART study): case-control study. Lancet.

[24] Tall AR, Yvan-Charvet L (2015). Cholesterol, inflammation and innate immunity. Nat Rev Immunol.

[25] Ridker PM, Everett BM, Thuren T (2017). Antiinflammatory therapy with Canakinumab for atherosclerotic Disease. N Engl J Med.

[26] Haniff HS, Knerr L, Liu X (2020). Design of a small molecule that stimulates vascular endothelial growth factor A enabled by screening RNA fold-small molecule interactions. Nat Chem.

[27] Vassiliadis E, Barascuk N, Didangelos A (2012). Novel cardiac-specific biomarkers and the cardiovascular continuum. Biomark Insights.

[28] Lin M, Jackson P, Tester AM (2008). Matrix metalloproteinase-8 facilitates neutrophil migration through the corneal stromal matrix by collagen degradation and production of the chemotactic peptide Pro-gly-pro. Am J Pathol.

[29] Dinarello CA (2018). Overview of the IL-1 family in innate inflammation and acquired immunity. Immunol Rev.

[30] Ridker, P.M., Macfadyen J.G., Thuren T., et al., Effect of interleukin-1β inhibition with canakinumab on incident lung cancer in patients with atherosclerosis: exploratory results from a randomised, double-blind, placebo-controlled trial. The Lancet. 2017;390(10105):1833–1842. 10.1016/S0140-6736(17)32247-X 28855077

[31] Li Y, Yao J, Han C (2016). Quercetin, inflammation and immunity. Nutrients.

[32] Geovanini GR, Libby P (2018). Atherosclerosis and inflammation: overview and updates. Clin Sci (Lond).

[33] Loke WM, Proudfoot JM, Hodgson JM (2010). Specific dietary polyphenols attenuate Atherosclerosis in apolipoprotein E-knockout mice by alleviating inflammation and endothelial dysfunction. Arterioscler Thromb Vasc Biol.

[34] Mulvihill EE, Assini JM, Sutherland BG (2010). Naringenin decreases progression of Atherosclerosis by improving dyslipidemia in high-fat-fed low-density lipoprotein receptor-null mice. Arterioscler Thromb Vasc Biol.

[35] Zeng W, Jin L, Zhang F (2018). Naringenin as a potential immunomodulator in therapeutics. Pharmacol Res.

[36] Orhan IE, Nabavi SF, Daglia M (2015). Naringenin and Atherosclerosis: a review of literature. Curr Pharm Biotechnol.

[37] Mayerl C, Lukasser M, Sedivy R (2006). Atherosclerosis research from past to present—on the track of two pathologists with opposing views, Carl Von Rokitansky and Rudolf Virchow. Virchows Arch.

[38] Grebe A, Hoss F, Latz E (2018). NLRP3 inflammasome and the IL-1 pathway in Atherosclerosis. Circ Res.

[39] Kanehisa M, Furumichi M, Sato Y (2022). KEGG for taxonomy-based analysis of pathways and genomes. Nucleic Acids Res.

