# Supplementary figures and images for: Anti-atherosclerotic effects of naringenin and quercetin from Folium Artemisiae argyi by attenuating Interleukin-1 beta (IL-1β)/ matrix metalloproteinase 9 (MMP9): network pharmacology-based analysis and validation
Source: BMC Complement Med Ther. 2023 Oct 25;23:378. doi: 10.1186/s12906-023-04223-1 (PMC10601115; doi:10.1186/s12906-023-04223-1)

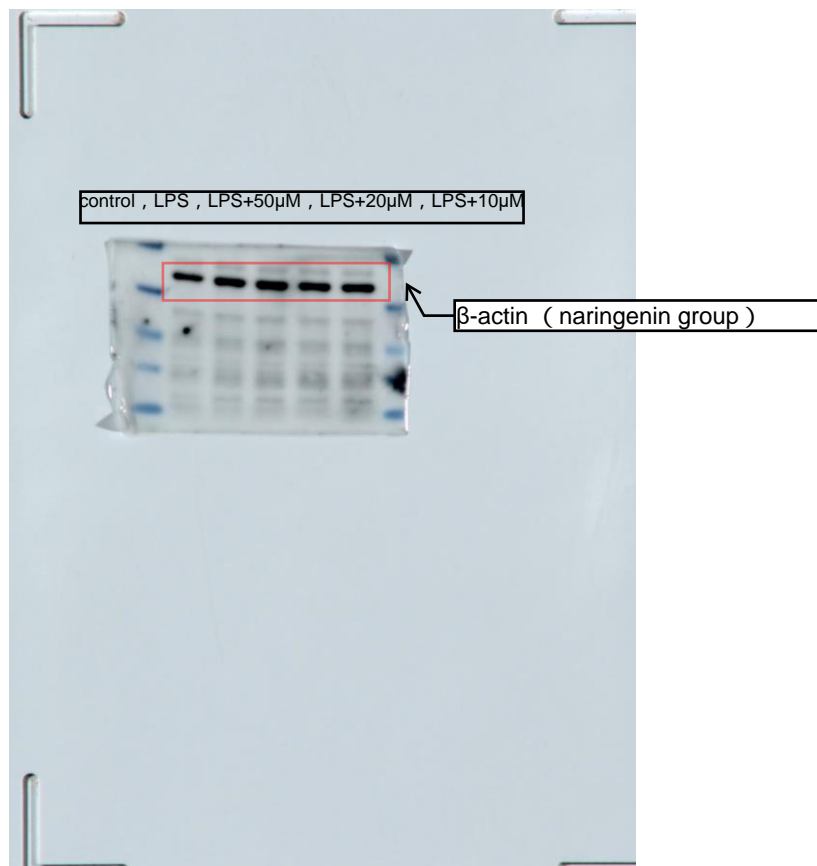

B-actin+Marker-Y

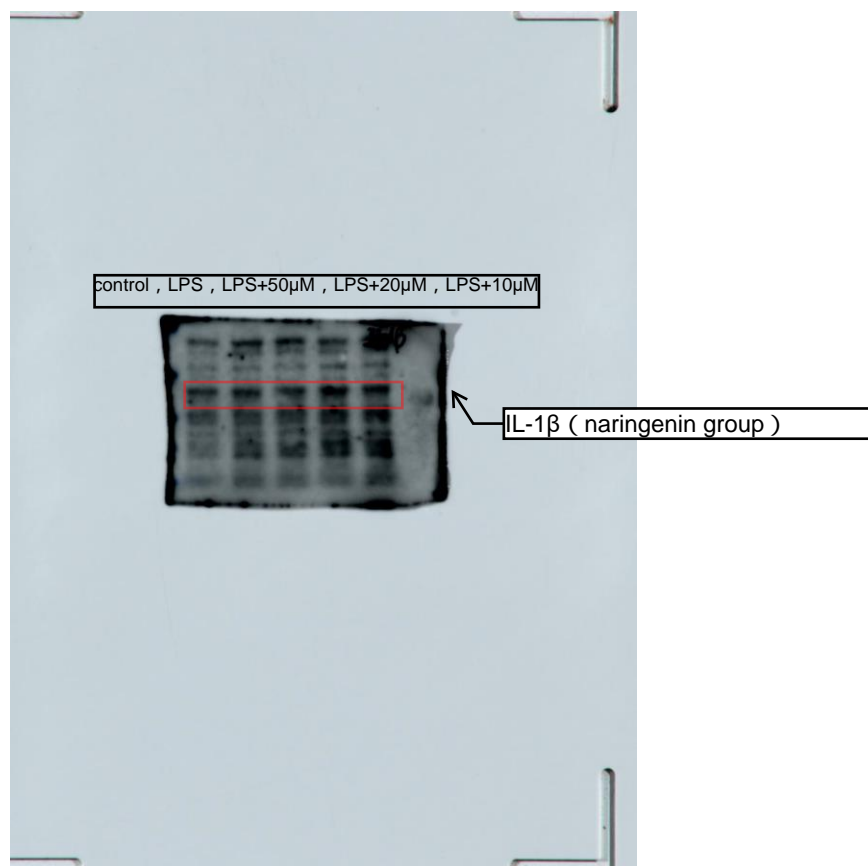

IL-1b+Marker-Y

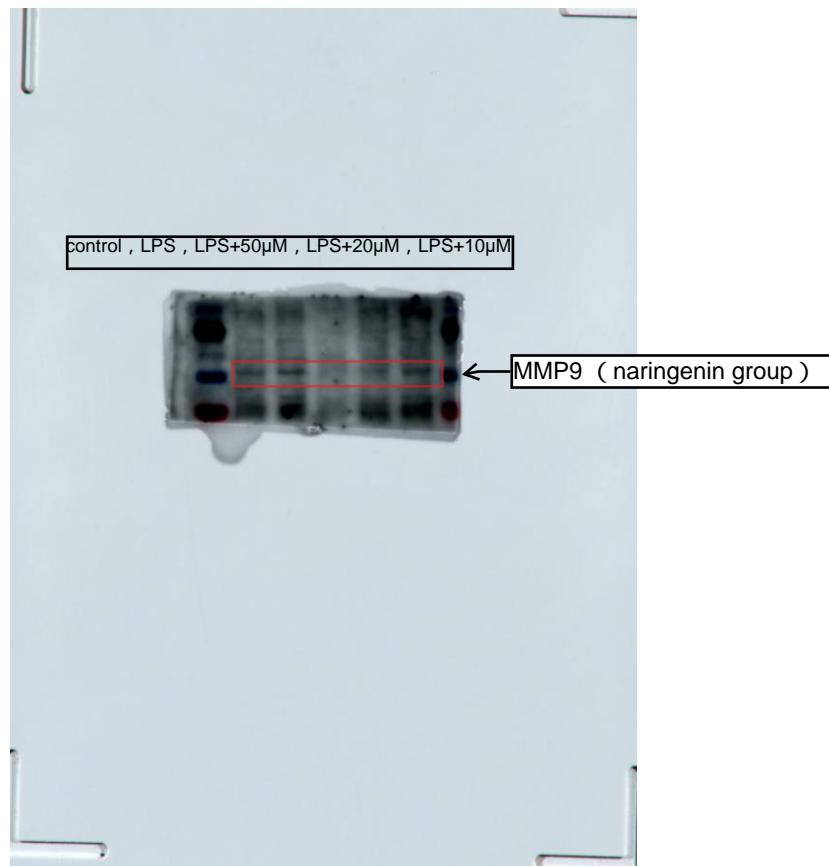

mmp9+Marker-Y

Supplement: Supplementary file 2 — Supplementary Material 2 [file 12906_2023_4223_MOESM2_ESM.pdf]

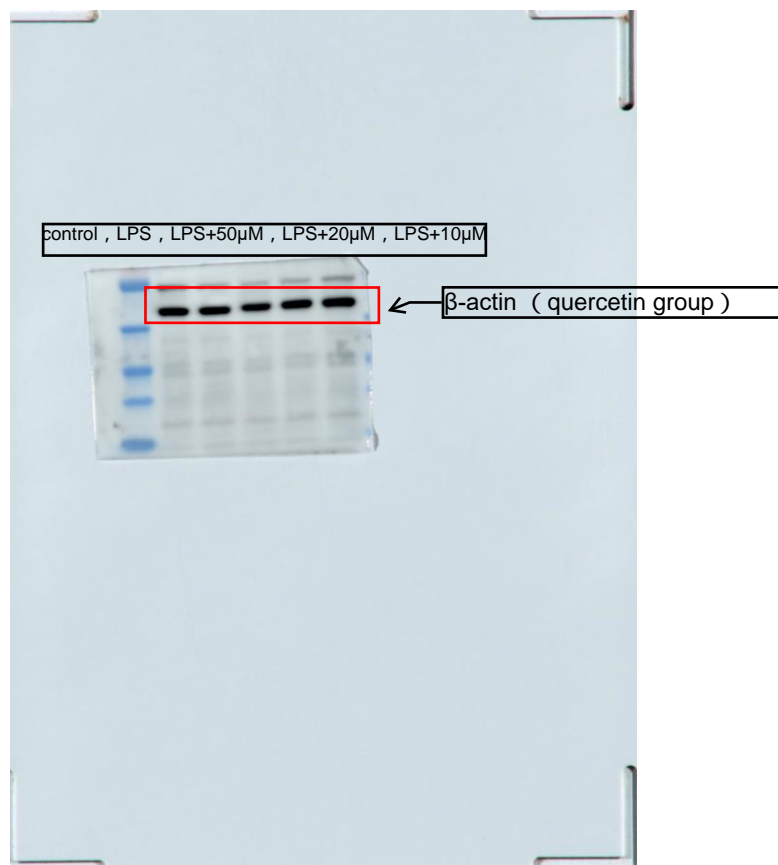

b-actin+Marker-H

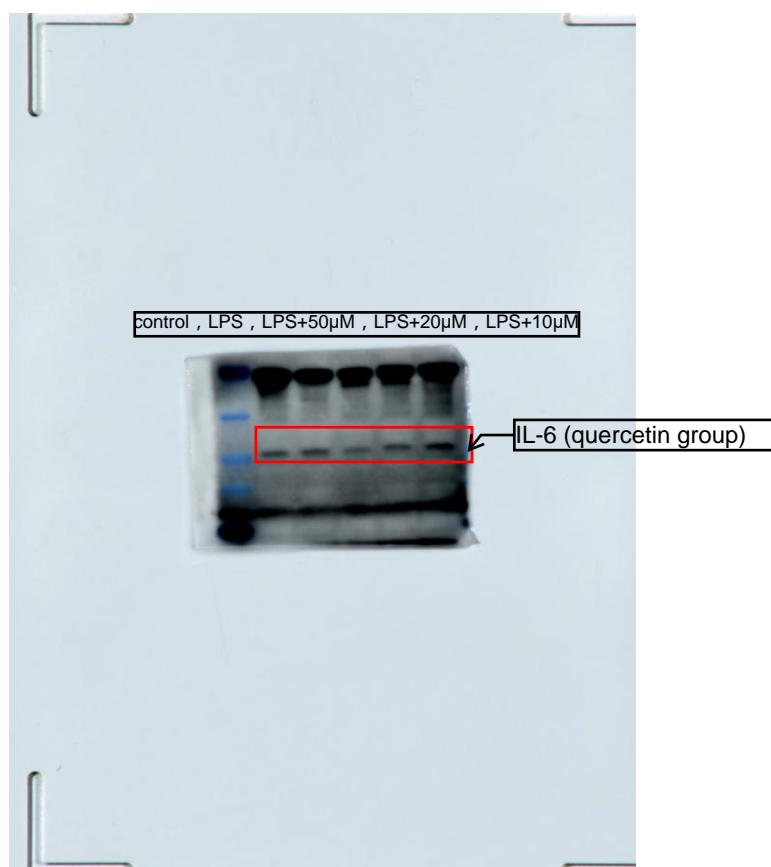

IL-6 +Marker-H

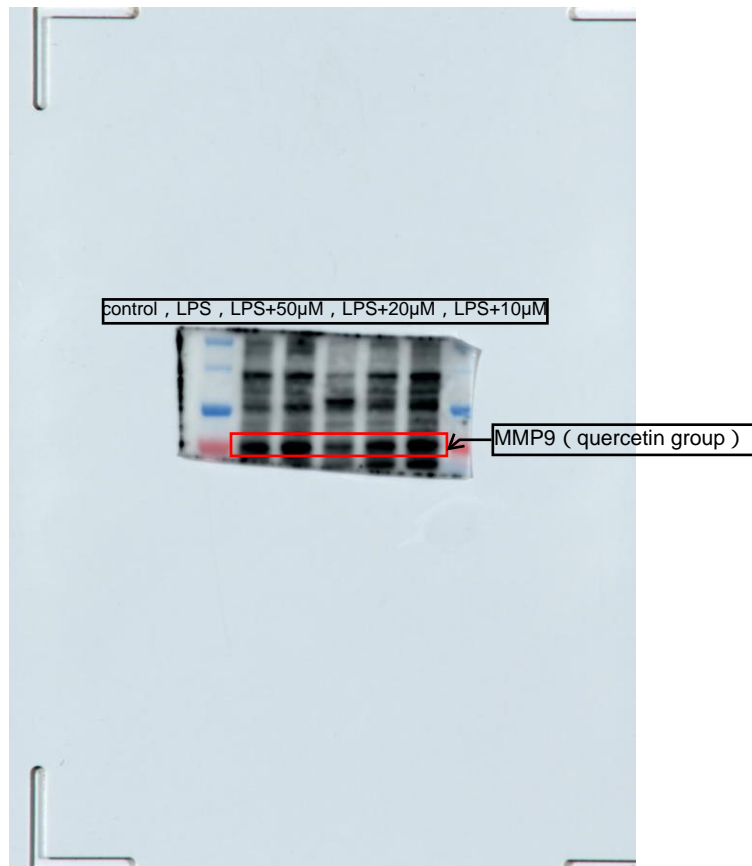

mmp9+Marker-H

Supplement: Supplementary file 3 — Supplementary Material 3 [file 12906_2023_4223_MOESM3_ESM.pdf]
